# Performance of theodolites versus drones in land-based studies of marine mammals

**DOI:** 10.1038/s41598-025-06978-8

**Published:** 2025-06-25

**Authors:** Thaya Mirinda Dinkel, Angélique Girard, Tom Bär, Michael Dähne, Ann-Kristin Craul, Mel Cosentino, Ole Meyer-Klaeden, Flemming Dahlke, Christian von Dorrien

**Affiliations:** 1 Thünen Institute of Baltic Sea Fisheries, Alter Hafen Süd 2, Rostock, Germany; 2 Institut Agro Rennes-Angers, 55 Rue de Saint-Brieuc, Rennes, France; 3 Deutsches Meeresmuseum, Katharinenberg 14-20, Stralsund, Germany; 4 Universität Rostock, Universitätsplatz 1, Rostock, Germany; 5 Aarhus University, C.F. Møllers Allé 3, Aarhus C, Denmark; 6 Institute of Marine Ecosystem and Fisheries Science, University of Hamburg, Große Elbstraße 133, Hamburg, Germany; 7 Present Address: Federal Agency for Nature Conservation, Insel Vilm, Putbus, Germany; 8 Present Address: Fleckeby, Germany

**Keywords:** Baltic Sea, Method comparison, Land-based observation, Odontocetes, Cetacean monitoring, Harbour porpoise, Behavioural methods, Animal behaviour, Behavioural ecology, Conservation biology, Ocean sciences

## Abstract

Theodolites and drones are key instruments for observing small whales in coastal areas. This study compared their performance while observing the harbour porpoise (*Phocoena phocoena*) in the western Baltic Sea. The methods were used simultaneously providing information on location, behaviour and group size during a field campaign in 2022. Theodolite observers were able to detect surfacing positions during 80.5% of porpoise sightings while a drone collected data during 50.7% of total sightings detected by plain eye. The drone footage quality was poor during 47.3% of these sightings. An in-depth analysis of 75:36 h of good quality footage resulted in 16:55 h (22.4%) of cetacean appearance. The determination of group size was significantly more precise using drone footage while the theodolite was more accurate in determining the start/end of a sighting. The accuracy of locations was modelled using the distance (D_t-d_) between recorded theodolite and drone coordinates of the same surfacing porpoise. D_t-d_ varied significantly based on the point quality. Sea state and porpoise to theodolite observer distance did not influence D_t-d_. Both methods complement each other and should ideally be used simultaneously to obtain both accurate and detailed information on harbour porpoises and other marine mammals during land-based observation studies.

**Supplementary Information:**

The online version contains supplementary material available at 10.1038/s41598-025-06978-8.

## Introduction

Observing cetaceans in their natural habitat is important and can serve many purposes. This includes investigating trends in abundance, creating a species inventory, assessing geographical and temporal distribution, mapping behaviours, understanding basic biology and life history parameters and conducting targeted experiments^1^. This information is not only useful for research but is also fundamental for scientific advice and decision-making on effective management measures^2^. Observing cetaceans in their natural habitat can nevertheless be challenging as the animals spend extended periods of time under water, exhibit unpredictable movement patterns with some species only briefly seen at the surface while covering vast distances^3,4^. A variety of visual, acoustic and other survey techniques can be used for this purpose. Visual observations can be land-based^4–6^, boat-based^7–9^, carried out with aircrafts or drones^7,10,11^ or using underwater cameras^12,13^. While their coverage is limited to the immediate vicinity, land-based observations are a popular method since they allow for a continuous monitoring of an area at relatively low costs without the risk of altering the natural behaviour of the species studied^3^. Land-based observation methods can be carried out from dedicated survey platforms or using platforms of opportunities such as light houses or other vantage points, ferries or oil drilling platforms to name but a few. Comparability of such different techniques is not always a given and suggests that comparison experiments are necessary to avoid biased conclusions from differently acquired data.

### Theodolites

Since their first use for marine mammal studies in the 1970s by Roger Payne, theodolites and total station theodolites (from here on referred to as theodolites) have been used to study at least 46 marine mammal species in 36 countries^3^. They allow recording geographical positions of marine mammals as well as collecting data on their behaviour^3^, distribution and relative abundance over time^14,15^. Some examples of their use are abundance estimates of humpback whales in east Australia^16^, the examination of detection radii of acoustic devices for harbour porpoises in Denmark^17^, estimating approach distances of harbour porpoises to a net and an acoustic alarm in Canada^18^, or to study the effectiveness of seal scarers as deterrent tools for harbour porpoises near construction sites^19^.

Theodolites are topographical equipment used in civil engineering and cartography to take precise (down to a few mm over hundred meters) measurements within a confined area, usually set up using official survey markers. A traditional theodolite measures only a vertical angle relative to the zenith, the position directly above the theodolite, and a horizontal angle relative to a reference object with known location and bearing from the theodolite^3^. A total station theodolite is an optoelectronic geodetic instrument that integrates the function of measuring angles with an electronic distance meter allowing to estimate distances from the instrument to a particular point and computing coordinates through the on-board computer^20^. Using the vertical and horizontal angles and the exact height of the station in relation to the variable sea level, the position of targeted object can be calculated based on trigonometric equations.

### Drones

Remotely piloted unmanned aircraft systems, commonly referred to as drones, have also become an essential tool for marine mammal observations allowing research on the movement, ecology, behaviour, health and habitat use as well as monitoring activities, during targeted experiments including minimally invasive cetaceans blow sampling or studying the effects of anthropogenic activity on marine mammal behaviour^7,21–25^.

The ability to observe animals from above is especially valuable for analysing behaviour of cetaceans that spend large amount of their time below but close to the water surface. Most drone platforms are equipped with various modules, such as Global Positioning System (GPS) receivers, accelerometers, magnetometers, ultrasound sensors and barometers, allowing for photogrammetric measurements from drones to produce results with small error levels. Drones thus expand the range of data that can be collected during land-based observations. Further, drones with high precision sensors are now available for a few hundred euros, making them accessible to everyone.

While drone technology has been adopted rapidly by researchers, there is still a shortage of studies that compare visual observations data derived from drones with that obtained by theodolite tracking. Furthermore, the possibility of combining both tools requires the consideration of data set comparability^26^ and identification of strengths and weaknesses of each method.

The aim of this study is to assess these two visual observation methods, reveal their shortcomings and analyse their comparability while studying the rather elusive harbour porpoise (*Phocoena phocoena*) in the western Baltic Sea. We present survey results obtained through a series of field trials and tested the following hypothesis: (1) accurate coordinates of harbour porpoise individual positions can be obtained from both methods, and at close distances they do not differ strongly; (2) drones allow identifying behaviour in more detail and detect formerly unknown behaviours accurately; (3) Visual data collected by both methods overlaps just partially, making the methods complementary rather than alternatives to each other. A SWOT analysis further gives insight into the scope of both methods. SWOT stands for Strengths, Weaknesses, Opportunities and Threats, and is a process in which the internal and external factors that affect a topic or entity are analysed for their performance^27^. Strengths and weaknesses are viewed as internal characteristics inherent to the methods itself, whereas opportunities and threats are external factors that may influence its success or performance depending on the environment^27^.

## Material and methods

### Study area

**Fig. 1:**
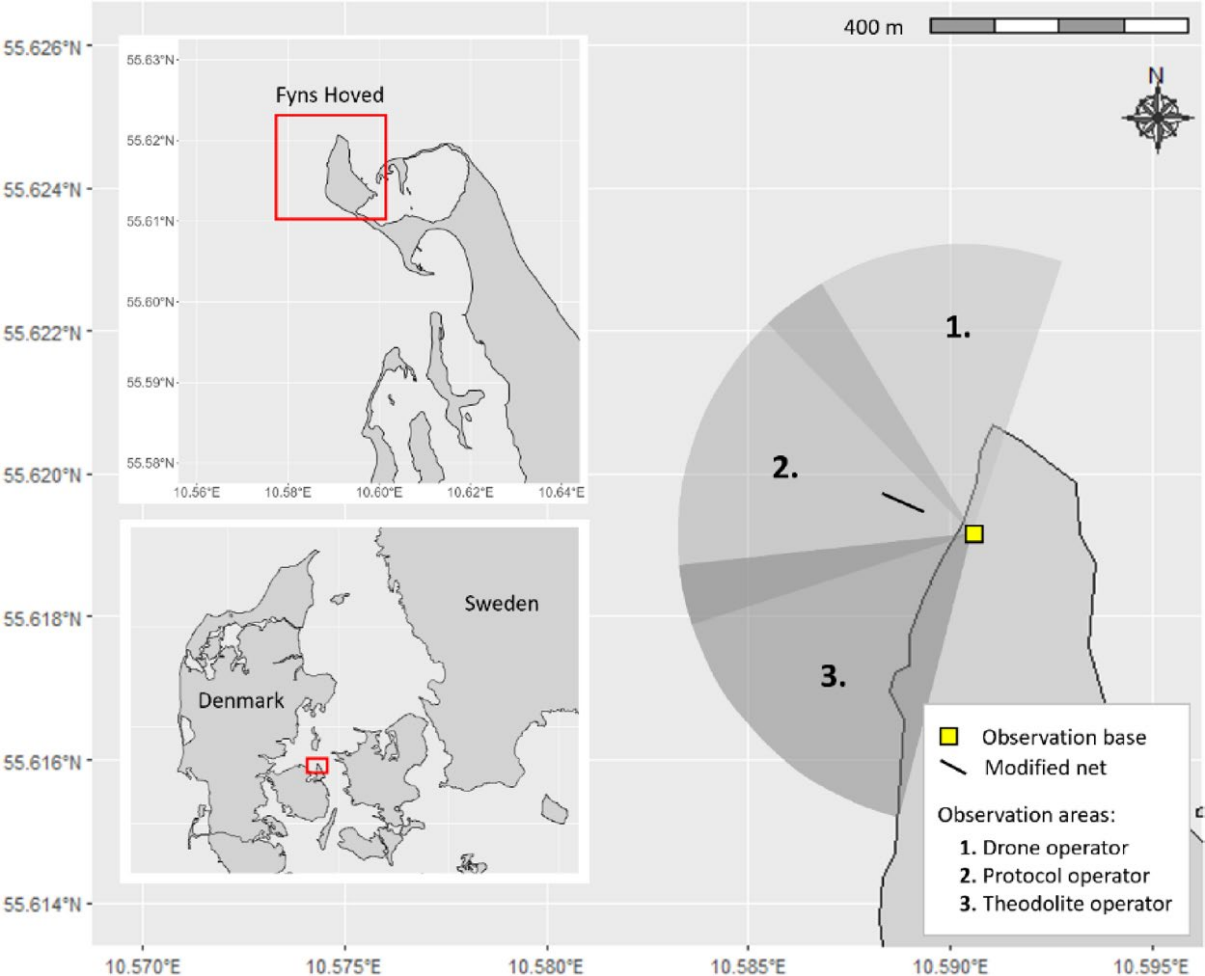
Survey area with positions of underwater equipment and three sections of observation by plain eye, with slight overlap (grey shadings 1–3). Each section was observed by an observer which also had additional specific tasks covering the entire observation area.

The study was carried out in the western Baltic Sea on the north-western side of the Fyns Hoved peninsula (Island Fyn, Denmark) from June 25 until August 28 2022. The data were collected as part of a study investigating behavioural responses of harbour porpoises to a temporally activated acoustic alerting device and an experimental net using acoustic and land-based observations. Three observers scanned the study area for porpoises by plain eye, each surveying a different section (north, central and south, Fig. 1). Each observer was also assigned a specific task—filling in protocols, operating the drone, or operating the theodolite – all covering the entire study area. Once a porpoise was sighted, the theodolite and drone operation started. At least three observers, but more often four observers worked at the same time. When four observers were present, two drones could be operated simultaneously: one stationary drone at 100 m above the take-off location filming the experimental set up with a ground resolution of 2 cm covering an area of approximately 82 m wide and 178 m long, and a following drone recording porpoises at lower altitude. Activities rotated every 30–40 min to add variety to the work hours, keep concentration up and allow an observer a break. Different observers on two shifts (< 6 h continuously) operated per day (max 10 h total) during daylight hours between 4am – 6 pm UTC. Field work was conducted with authorization from the Danish Nature Agency (Naturstyrelsen).

### Recorded data

A sighting was defined as at least one porpoise or group surfacing at least two times seen by two observers. An event ended when no porpoise was seen for 10 min. An additional (new) porpoise or group sighted during an on-going event was recorded as a new sighting. When several individuals mixed, it was not always possible to tell which porpoise belonged to which sighting, and the observation was marked as a merged sighting in the protocols. During a sighting event all observers turned to recording data, take theodolite points and flying the drones (Fig. 1). Observers were asked to briefly scan the area in between individual tasks if time permitted. The time, number of porpoises, general behaviour, initial heading direction, theodolite points, drone activity and the weather were recorded in different protocols (protocol tables as supplementary material). Observations were interrupted or terminated when sea state was higher than 3 which corresponds to a wave height between 0.5 to 1.00 m.

### Theodolite

The Leica Flexline plus TS06 + total station theodolite was used for the tracking of surfacing porpoises from a stationary point on a 20 m cliff. Exact cliff height was measured by stationing the theodolite using three permanently marked large stones with known location on the beach. The height of the stones was calibrated against a sea level meter that was installed behind the cliff in a small lagoon, where wind did not interfere with reading the variable sea level using the theodolite. Horizontal and vertical angles were used to compute the geographic position of the targets. From here on, the computed positions in UTM will be referred to as theodolite points.

The target for the theodolite cross hair when using the ocular was the centre of the animal or its footprint (surfacing turbulences). On some occasions the secondary crosshair on top of the theodolite was used to collect points faster resulting in less accurate positions. All possible surfacing individuals were recorded to get an impression of the distribution of the group. If a group of porpoises split up, the theodolite operator concentrated on the porpoises that the drone operator was following. Most observers had no previous experience using a theodolite but had a training session during one day before collecting data in the field. The quality of a point taken was ranked as Q1) porpoise/footprint seen exactly at measured position; Q2) tracking point close to last surfacing; Q3) tracking point was taken as an approximation of the last surfacing point. For each measurement, the observed behaviour, swimming direction and quality were noted.

### Drones

Three DJI Mini 2 and one Air 2S drones (www.dji.com) were used. Drones were operated using the Litchi App (www.flylitchi.com) and flightlogs were automatically synchronized to www.airdata.com. Drones were controlled using either shaded android smartphones or an apple tablet attached to the controller.

The following drone was used for detailed focal follows^28^ of porpoises. A stationary drone was flown 100 m above take-off location and centred over the experimental set up to record reactions of porpoises towards the net. After July 28th, this drone was continuously in position flying a new drone into position once the battery level was low, to avoid missing net interactions. Theodolite and following drone operator were in constant exchange to agree on the individuals to follow. From July 11th on, drone operators assessed the quality of the recorded following drone videos to provide information on the recorded files and to facilitate post-hoc analyses of the videos. Drone footage quality was ranked as D1: porpoise is on the video nearly full time; D2: porpoise can be seen on the video but with interruptions; D3: porpoise can be seen on the video just for a few seconds or not at all. The altitude of the following drone ranged between 10–90 m above the water surface. Drones were flown during good weather conditions, i.e. no or little precipitation, no fog and wind speed < 10 m/s. The observed sea state and the monitored wind speed were not always in direct relation as the cliff shielded the study area during easterly winds. Flight duration was limited by battery capacity which was about 20–25 min for both drone models. A lead acid battery and inverter provided charging for drones, phones and controllers. All users operated the drones under the possession of a drone flying certificate, issued by the European Union Aviation Safety Agency for the A1/A3 open sub category.

### Video analysis

Drone footage was screened after the field campaign. Mostly following drone videos with quality D1 and D2 were assessed. Those videos were watched at normal speed and played back when necessary. A newly developed software, CetTrack was used to extract geographic positions of porpoises in the footage. The software uses the flight log data (date, time, latitude, longitude, altitude of the drone above medium sea level, heading direction and gimbal orientation), drone data (aperture angle of the camera, and number of pixels of the image recorded) and subtitle information for synchronization purposes.

### Comparison data collected by both methods

The number of theodolite points recorded during a sighting was compared with the quality of the following drone footage for the respective sighting pairs. A Kruskal–Wallis test was performed for 278 sighting events. The stationary drone footage was not included in this analysis as the drone flew continuously on the same position independently if there was a sighting going on or not. All positions from following drones and theodolites were taken into account for producing kernel density plots^29^ of the location data using the libraries ggpubr^30^ and eks^31^ under R version 4.3.1^32^.

### Group size

The estimated porpoise group size recorded by the theodolite observers was compared to the number of individuals observed during the screening of the drone footage for the same sighting played at normal speed. Following drone footage quality D1 and D2 were prioritized as well as stationary drone footage in which porpoise presence was marked in the protocols. 80 sightings were analysed corresponding to 75:36 h of drone footage. A Wilcoxon signed-rank test was used to compare the group size counts.

### Behaviour

The behaviour recorded by the theodolite operator on the cliff was compared to the behaviour that was observed in simultaneously taken drone footage. For this purpose, two exemplary videos were selected. Videos were selected after initial screening and choosing sightings in which at least 20 theodolite points were taken. The time of occurrence of each individual visible in the footage was noted and each individual was assigned an ID. Sex and age were classified based on the body size and the swimming distance between individuals. Two porpoises with significantly different body sizes swimming very close to each other were classified as a mother-calf pair (MCP); assuming that the bigger individual is the mother and the smaller is the calf. This was verified by observed suckling events. Male porpoises were differentiated from other individuals by observed mating attempts in which the penis was visible. Five general behavioural categories (travelling, feeding, socialising, not classified and not visible) were established based on definitions from previous studies and a larger behavioural analysis for the area^33^. The exact time stamps for each observed behaviour and surfacing event in the video were recorded for each porpoise that appeared in the footage. All behaviours observed by the theodolite operator and the video analysts were plotted together to allow for comparison.

### Drone and theodolite metrics comparison

Porpoise coordinates were extracted from drone footage to be compared to coordinates collected simultaneously with the theodolite. For this aim 22 sightings encompassing 6:18 h of drone footage were tracked. Sightings were prioritized based on two criteria: 1) single porpoises or MCPs in close proximity to allow for an easy matching process; 2) sightings with more than 20 theodolite points. For MCPs, the surfacings of the mother were taken as coordinates for matching. The entire sighting was not always tracked as in some occasions more porpoises joined the sighting which prevented clearly matching theodolite and drone coordinates. In total, 153 coordinate pairs could be matched using the date and time allowing for a maximum delay of 30 s between the drone footage and surfacing theodolite point to account for the time takento manually aim and measure surfacings. Once pairs of drone and theodolite coordinates were identified, the Euclidian distance between the UTM coordinates (D_t-d_: Distance theodolite − drone) was calculated.

D_t-d_ was used to test if the accuracy of the theodolite coordinates varies with the distance to the instrument, the sea state and the self-assessed theodolite point quality using a generalized linear model (GLM) with Gamma distribution (log link). The variables were reduced by choosing the best model fit selecting the lowest AIC value. The accuracy of the coordinate position of the drone is affected by the satellite uplink (number of satellites that are in direct line of sight to the drone), and the availability of the different Global Positioning Networks (GPS, GLONASS and GALILEO). The DJI mini 2 drone has a vertical hovering accuracy of ± 0.5 m and a horizontal accuracy of ± 1.5 m (both with GPS Positioning, DJI Mini 2 characteristics, 2023). Precision of the coordinate estimates will vary furthermore with camera characteristics such as gimbal angle, movement (abrupt, slow), mode of operation (P-GPS, “Sport-mode”, etc.), and if the animals are in the centre of the frame or more towards the edges of the image. In the field, drones were usually started when the drone indicated that it had enough satellites to establish a stable GPS connection. Drone footage was tracked when the gimbal was near 90° while avoiding taking points after abrupt movements or rotations in the footage. The precision of the absolute position estimates of the drone is around 2 m after the first assessment during calibration trials.

### Time and distance

The time of the first and last theodolite and drone coordinate during the same sighting were compared to assess if one method is better at capturing the initial or end phase of a sighting event. The distances of the first and last coordinates recorded with both methods from the observation site were also calculated for the 22 sightings (Table 3). A linear correlation using a square root transformation was performed to explore if the distance data provides a pattern. Sightings with fission and fusion events^34^ were excluded for this analysis due to unclear start/ending times and positions. All analysis were conducted using R version 4.3.1^32^.

## Results

576 harbour porpoise sightings were recorded by plain eye, some of them on nearly all observation days (Table 1). The mean number of sightings of porpoises per day varied, with more sightings in August than in July.

**Table 1:** Summary of field effort collected by different visual observation methods.

		Total	June	July	August
Sightings	Nr of days with sightings	44	3	16	25
	Total nr of sightings	576	12	159	405
	Mean Nr of sightings (NS) per day	13.1	4.0	9.9	16.2
Theodolite	NS with theodolite points (TP)	464	8	109	347
	NS with ≤ 3 TP	163	5	42	116
	NS with ≥ 4 to 10 TP	141	1	41	99
	NS with ≥ 10 TP	160	2	26	132
Drone	NS with 100 m drone*	476	1	97	378
	NS with following drone	292	2	65	225
	NS D1**	89	-	3	86
	NS D2**	36	-	12	24
	NS D3**	138	-	42	96
	NS no quality ranking	29	2	8	19

*Stationary drone flown continually after July 28th. **Following drone footage ranking introduced on July 11th.

### Theodolite

**Fig. 2:**
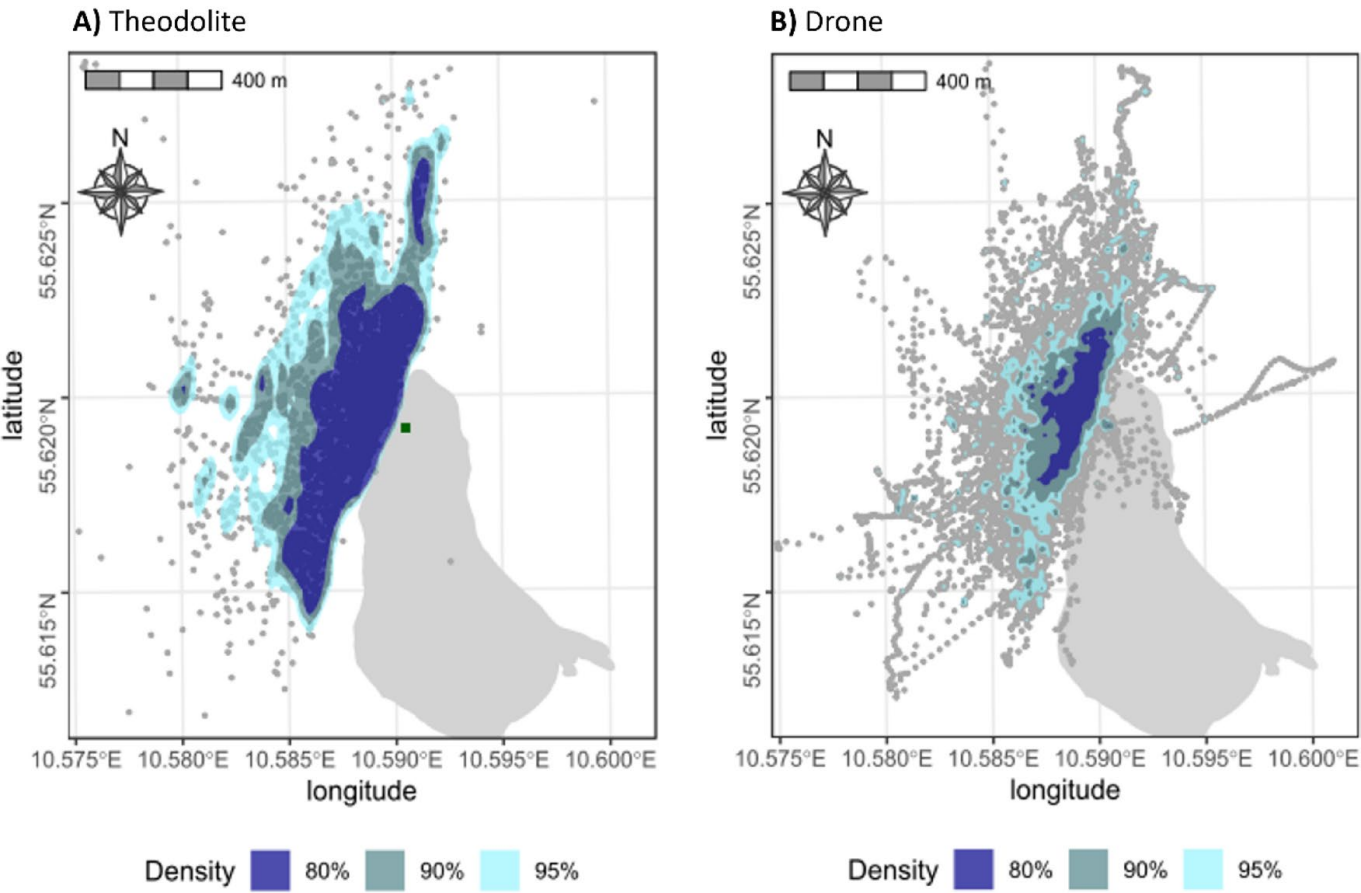
Kernel density plots illustrating (**A**) the distribution of theodolite points in the study area; (**B**) density of drone effort, based on all flight tracks. Note that drone tracks occasionally extend over land due to flight paths taken during repositioning or return to base. Core observation areas include 80% of all theodolite/drone locations (dark blue) 90% (grey) and 95% (light blue) respectively.

At least one theodolite point was recorded for 80.5% of the sightings, with a total of 5 058 points taken during the field season. The closest point from the instrument was taken at 55 m while the furthest point was taken at 1.4 km distance. All three self-assessed qualities of theodolite points are found throughout the area in which the data were collected (Table 2). Points are distributed near the coast, with more locations taken towards the north and south compared to the west (Fig. 2A.). 66.2% of the points were taken closer than 300 m from the instrument.

**Table 2:** Number of theodolite points taken according to distance from the instrument and classified by quality.

Distance from Theodolite (m)	Nr points taken	% of total	Quality of points		
			Q1	Q2	Q3
< 150	1488	29.4	669	599	218
150–300	1860	36.8	559	787	507
301–450	792	15.6	230	265	292
451–600	531	10.5	240	143	146
601–750	257	5.1	163	50	43
751–900	79	1.6	30	36	13
> 900	51	1.0	29	15	6
Total points	5058	100	1920	1895	1225

### Drone footage

248 h of drone footage were recorded during the field campaign. Drone effort was concentrated near the coast with the kernel densities covering a smaller area compared to the theodolite points, but showing the same general distribution pattern with more drone locations in coastal areas north and south compared to the offshore areas in the west (Fig. 2B).

### Video analysis

75:36 h of D1 and D2 quality footage were screened. Porpoises were seen during 16:55 h within these videos, corresponding to an appearance rate of 22.4%. Visualizing a 20-min video and filling in the corresponding data table took between 25–45 min as videos had to be paused frequently to confirm a sighting or differentiate between a wave movement, glare, a bird or seal or re-find animals that had submerged. Twenty-two sightings were selected for tracking to extract coordinate positions of harbour porpoises (Table 3). Four sightings were tracked taking a coordinate every 0.5 s to showcase how detailed the resolution of the movement pattern can be captured with the drone. The remaining videos were tracked taking points only when the porpoises surfaced to compare those coordinates to the theodolite coordinates.

**Table 3:** Tracking times and results for drone footage in comparison to the theodolite.

#	Date (2022)	Sighting ID	Drone type DJI	Total flight duration (mm:ss)	Time tracked in video (mm:ss)	Number of animals in video	Approx. tracking duration (min)	Tracking interval	Nr of tracked surfacings in drone footage	Nr of matching points (theo – drone)	ΔTime 1st point taken vs start of sighting (mm:ss)		∆Time last point taken vs end of sighting (mm:ss)		Distance to base, first point (m)		Distance to base, last point (m)	
											Theod.	Drone	Theod.	Drone	Theod.	Drone	Theod.	Drone
1	26.08	21	Mini2	33:02	12:59	2	240	0.5 s	39	14	Several sightings merging							
2	26.08	19	Mini2	33:09	10:15	2	210		37	10	Several sightings merging							
3	27.08	9	Mini2	20:51	01:20	2	40		18	7	00:54	28:28	No end time	298.7	279.8	87.2	128.0	
4	16.08	26	Mini2	21:26	10:36	2	60	Surface	26	5	01:31	02:53	03:28	06:21	159.5	103.2	64.2	173.6
5	21.07	3	Mini2	19:20	01:18	1	20		5	1	17:20	16:54	00:00	08:23	NA	32.2	115.6	16.0
6	26.08	11	Mini2	09:50	01:27	1	20		4	2	00:53	03:20	01:59	03:14	184.1	366.3	407.3	361.0
7	30.07	3	Mini2	13:41	02:52	1	30		7	5	01:15	03:34	00:51	07:47	201.5	218.0	805.3	209.9
8	22.08	15	Mini2	21:30	04:14	1	50		12	7	01:06	04:12	04:19	09:32	191.5	414.3	770.3	527.4
9	04.08	3	Air2S	14:16	04:48	2	60		27	5	03:09	02:04	00:36	04:32	208.2	208.6	528.9	341.1
10	08.08	13	Mini2	13:38	02:00	1	30		5	3	01:26	05:37	00:41	03:26	90.5	441.0	24.7	269.4
11	14.08	4	Mini2	14:35	02:20	2	20		8	2	00:09	04:56	01:02	00:00	118.6	155.6	185.0	295.3
12	14.08	5	Mini2	12:41	04:05	2	40		27	15	00:20	03:01	04:21	03:15	927.3	314.7	299.3	307.6
13	26.08	5	Mini2	15:09	09:03	3	40		60	18	00:12	03:02	00:00	03:35	496.1	241.4	115.3	101.0
14	27.08	4	Mini2	20:39	09:58	2	40		46	14	00:00	01:29	03:50	16:35	285.0	100.0	109.2	49.5
15	25.08	5	Mini2	17:33	05:55	2	50		30	7	00:00	02:47	01:55	02:29	25.1	285.5	456.2	602.1
16	25.08	9	Mini2	19:51	15:00	2	60		48	15	01:10	02:46	01:54	13:54	169.9	85.0	164.1	187.7
17	22.08	5	Mini2	18:44	08:23	5	40		59	3	01:40	03:30	00:06	05:14	170.8	72.3	387.2	61.4
18	20.08	7	Mini2	21:37	03:27	2	90		23	5	01:37	11:30	10:50	19:36	648.8	302.7	663.2	389.4
19	20.08	11	Mini2	20:08	01:47	4	30		8	2	Several sightings merging							
20	22.08	7	Mini2	20:38	00:56	2	50		8	4	01:06	04:12	02:05	02:14	202.8	246,4	375.1	385.4
21	15.08	11	Mini2	07:32	03:52	2	45		14	3	01:51	03:25	No end time	289.5	117.0	63.2	134.6	
22	26.08	8	Mini2	09:08	00:33	2	30		5	1	07:07	22:36	01:46	04:58	385.1	430.5	506.8	409.5
Mean (min)				18:08	05:19	2.05					02:15	05:22	02:20	06:46				
Median (min)				19:02	03:59	2					01:10	03:25	01:54	04:58				
S.D. (min)				06:28	04:17	0.53					03:58	05:36	02:37	05:23				

### Comparison of data collected by both methods

**Fig. 3:**
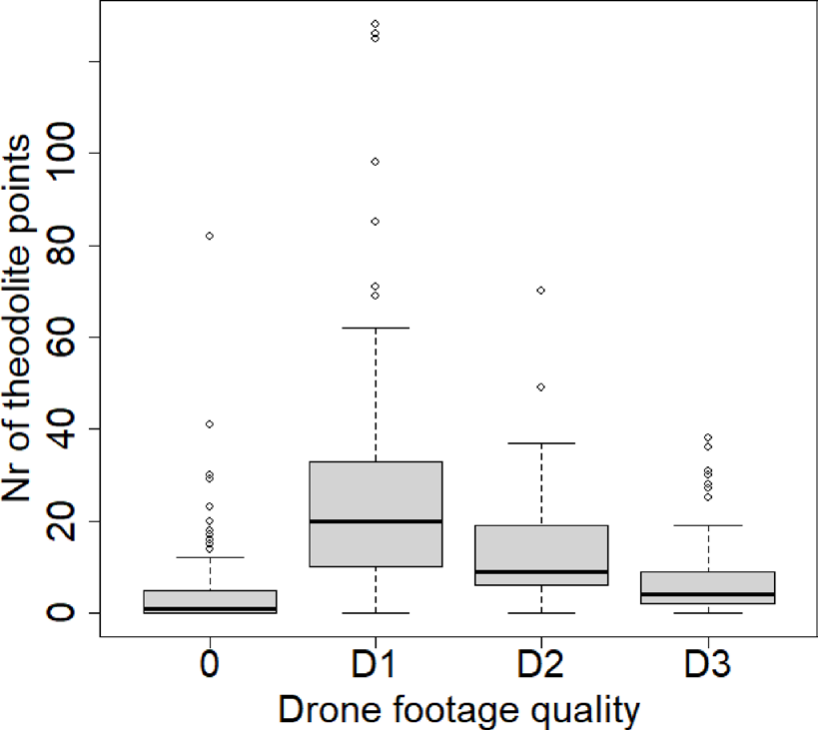
Drone footage quality and availability of footage in relation to the number of theodolite points taken during a sighting.

Theodolite points were taken during 80.5% of the sightings while the following drone was started during 50.7% of the sightings. Significant differences were found in the number of theodolite points taken during flights with different drone footage quality (Kruskal–Wallis test: p < 0.0001). A Dunn-Bonferroni post-hoc test was performed to identify differences between quality categories. Sightings in which the video footage was classified as D1 (mean: 26.6 points, SD: 26.9; min: 0, max: 128) showed no significant differences to D2 footage (mean: 14.0 points, SD: 14.5, min: 0, max: 70) (adjusted p > 0.05), but a significantly higher number of taken theodolite points than D3 footage (mean: 6.8 points, SD: 7.9, min: 0, max: 38) (adjusted p < 0.05). D2 footage also showed significantly higher number of theodolite points taken than during D3 footage (adjusted p < 0.05). Sightings in which the following drone was not flown had a mean number of 3.9 theodolite points (SD: 7.4, min: 0, max: 82) and was significantly lower than all the other drone footage qualities (adjusted p < 0.05) (Fig. 3.).

### Group size

The group size of the porpoises observed by the theodolite operator ranged from one to six porpoises and was significantly smaller than the counts obtained by analysing the drone footage—between one and eight porpoises—for the same sighting (Wilcoxon Signed-Rank Test: p = 0.023). Median difference in number of porpoise counts indicated no difference, while the mean showed an underestimation of 0.25 (SD: 1.06) individuals per sighting with the theodolite.

### Time and distance

The 22 sightings for which drone coordinates were extracted (Table 3) indicated that the theodolite provided first detailed information (smaller difference between start of sighting and first point taken) and remained longer with the porpoises (smaller time difference between the end of sighting and last taken point), compared to the drone coordinates. The distance from the observation station at which the first theodolite point and first drone coordinate was taken varied for most of the sightings without a clear visible pattern (linear regression R = 0.018, p = 0.596, Table 2). The final points however show a clearer relationship (R = 0.387, p = 0.004), with theodolite points being further away than drone points (Supplementary material).

### Local distribution data

**Fig. 4:**
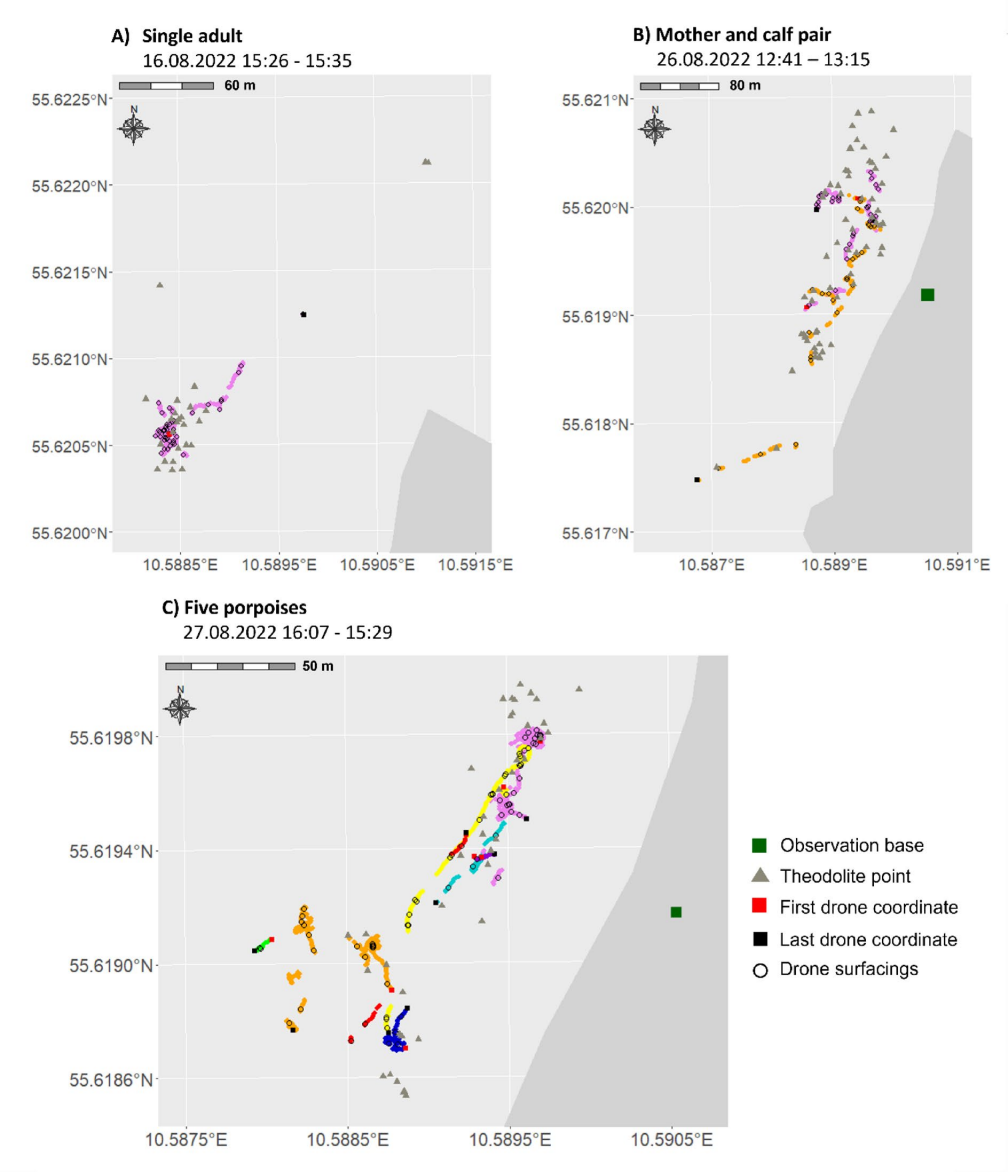
Porpoise drone tracks and coordinates from the theodolite for (**A**) single adult porpoise recorded during one flight, (**B**) a MCP recorded during two flights (pink: first flight, orange second flight), (**C**) five porpoises recorded during one flight. Each porpoise ID (8) is marked with a different colour.

Points collected with the theodolite give general information on the location of the porpoises in the study area highlighted by three examples in Fig. 4. Theodolite starting points are more accurate in defining where the sighting started than the drone which must first be started and generally takes some time until it finds the porpoise. On some occasions the theodolite operator was also able to follow the animals for longer periods and at further distances than the drone, as can be seen in Fig. 4A where only the theodolite operator tracked some points that indicate a northward movement of the porpoise. In Fig. 4B a gap in drone recordings occurred when a sighting was longer than 20 min (drone battery capacity limit). In this case, theodolite points could be collected continuously while one drone had to be flown back and a new one started, thus missing a part of the sighting in the northern part of the map. Drone footage nevertheless allows collecting very precise distribution information of the animals especially under the water surface which is missed by the theodolite, as can be seen in all graphs. It was feasible to track several animals during one sighting using the drone footage (Fig. 4C). Nevertheless, drone tracks are often interrupted which occurs when the porpoise dives deep, its colour merges with the sea floor or the drone operator loses it. Once an individual is lost or exits the frame, it is often not feasible to determine if the same porpoise is seen again in a group once it re-enters the video frame. This prevents a continuous observation of the same individual especially in larger groups where not all the porpoises can be followed at the same time as they often spread out or move into different directions. This is the case in Fig. 4C where 5 porpoises were present during the sighting, but 8 different IDs had to be attributed to the individual tracks as it was not possible to reassign IDs to the porpoises that re-entered the video frame.

### Behaviour

**Fig. 5:**
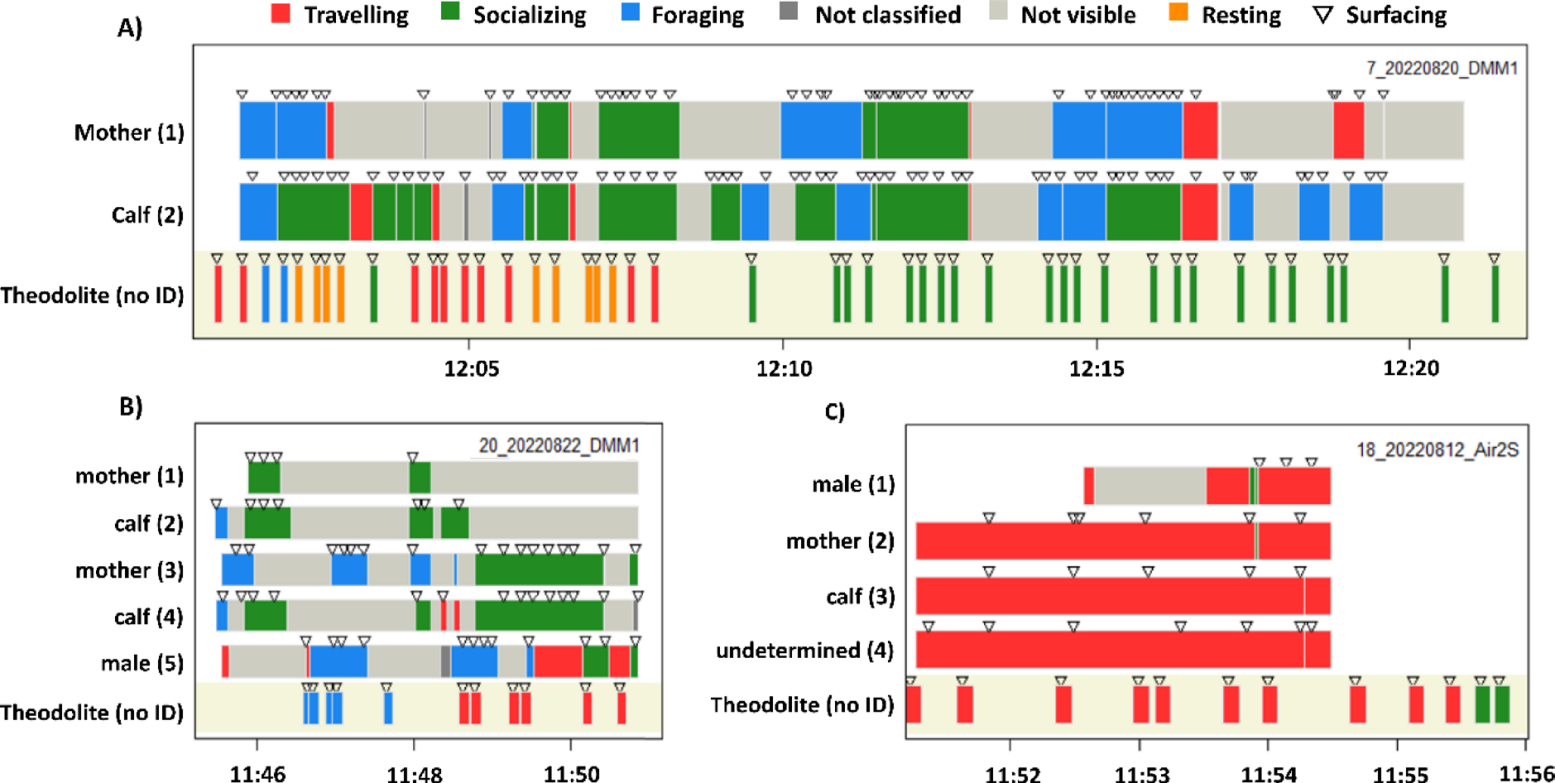
Observed behaviour of porpoises in drone footage and simultaneously taken theodolite points of (**A**) Mother and calf pair; (**B**) two mother and calf pairs and a male porpoise; (**C**) four porpoises (see text for further explanation).

While general behaviour observations were recorded by the theodolite observer, these behavioural categories did not always match the drone footage (Fig. 5A, B). Further, surfacing events do not align neatly, with theodolite points always being recorded with some delay, probably due to the time that an observer needs to see the animal, adjust the theodolite and take the point and notes. More surfacing events were recorded in the analysed drone footage compared to the theodolite points taken in the field. Figure 5A shows a MCP that was foraging, travelling and socializing. Behaviour categories attributed by both methods do not match, especially not during the first nine minutes of the tracked period. The theodolite operator indicated socializing during the rest of the sighting, as the event occurred within a MCP. In this context, a degree of social interaction can be assumed, given that the animals consistently remained in close proximity to one another. The drone was nevertheless able to discern more concrete behaviour within this interaction such as foraging intervals and travelling events which were not identified by the theodolite operator. This figure further shows an entire drone flight of around 20 min. The theodolite operator took points already before the drone operator had found the animals and also for a longer period as the drone had to fly back limited by drone battery capacity. Figure 5B shows a group of five porpoises that were close together so that they could be captured in the same drone frame. Behaviour recorded with the theodolite aligns better in this case but is representing all the surfacing events of the five porpoises without being able to differentiate between the porpoise individuals. Analysing the drone footage allows attributing individual behavioural categories to each of the five porpoises individually. Figure 5C shows an example where the behaviour recorded by the theodolite operator matches the drone. Theodolites can thus in occasions provide reliable behaviour information for groups, when all individuals show the same behaviour. Socializing events or assigning MCP cannot be achieved with the theodolite yet can be detected in the drone footage. However, also the drone is unable to give full track information as porpoises are often not visible in murky water. All figures show that there are regular gaps in the observation.

**Fig. 6:**
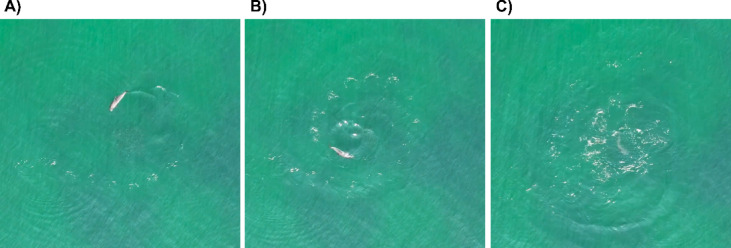
Individual harbour porpoise herding a school of small fish. (**A**) School of fish visible in centre; (**B**) close bordering of school (**C**) diving down in the centre (potential hunting attempt).

Drone footage also revealed that some behaviours are carried out exclusively underwater and therefore could not be detected by the theodolite operator. One special behaviour was observed in several occasions of an individual harbour porpoise herding a school of small fish (Fig. 6), potentially sprat, which has only been described as a group hunting strategy so far^35^.

### Modelling location differences

D_t-d_ was calculated for 153 coordinate pairs. The theodolite points that were available to match with drone positions consisted of 83 quality 1 (Q1) theodolite points, 39 quality 2 (Q2) coordinates, 31 quality 3 (Q3) points and were taken from a distance of 82 to 465 m from the instrument.

None of the interactions in the GLM analysis were statistically significant in explaining the variability in D_t-d_ as well as sea state and distance from the instrument (AIC of full model: 1076.3). Only the theodolite quality showed a statistically significant effect in explaining variability in D_t-d_. A reduced GLM was performed including only this factor resulting in a lower AIC of 1066.8.

$${\text{log}}\left( {{\text{D}}_{{{\text{t}} - {\text{d}}}} } \right) = \beta 0 + \beta {1} \cdot {\text{theo}}\_{\text{quality}}$$

**Fig. 7:**
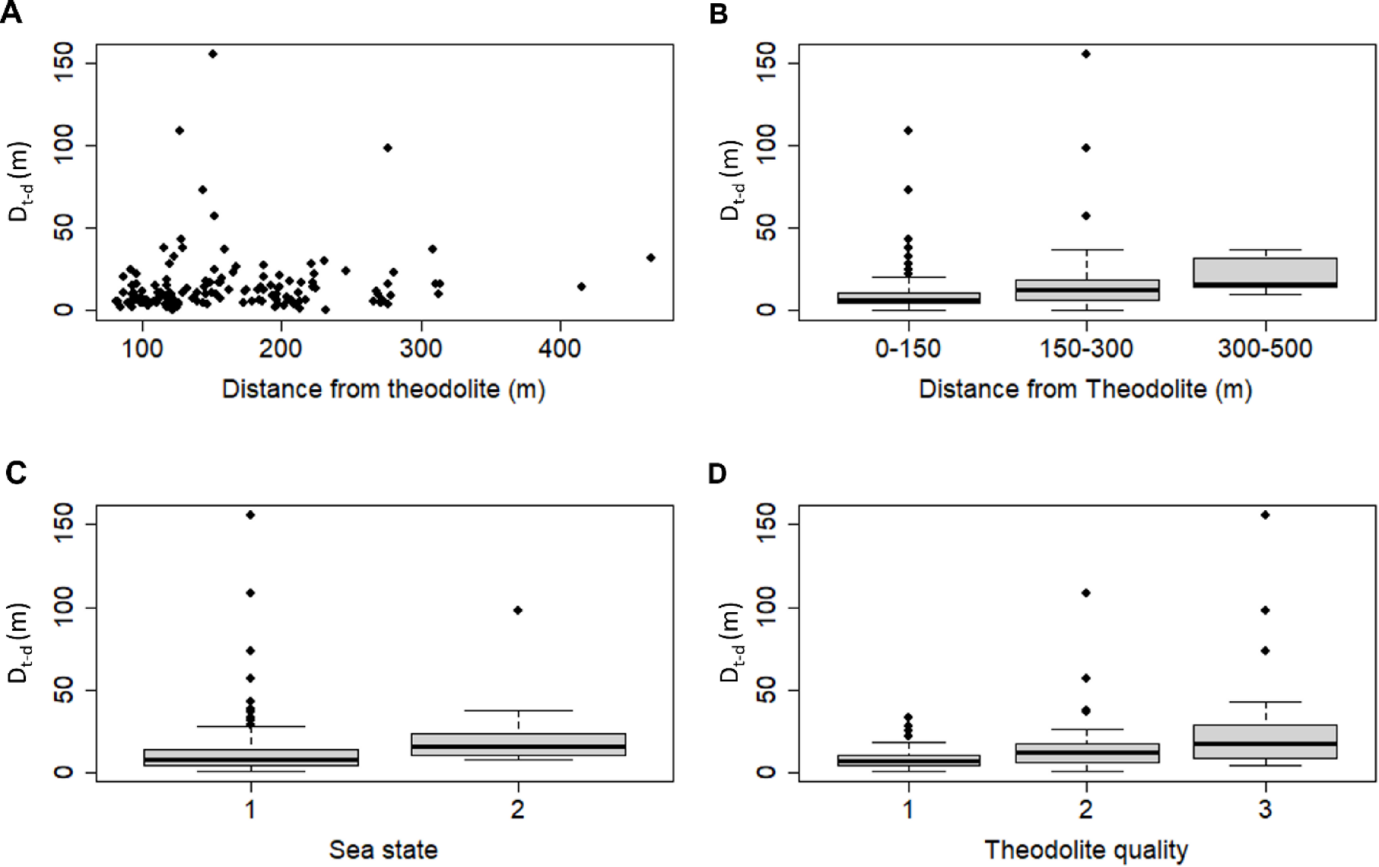
D_t-d_ in relation to (**A**) Distance from the theodolite; (**B**) distance categories from the theodolite; (**C**) for Sea State 1 and 2; (**D**) for the three theodolite quality categories.

D_t-d_ was significantly lower for Q1 points (mean: 8.3m, min. 0.4, max. 37.7m, s.d.: 5.99m) compared to Q2 points (mean: 16.3m, min. 0.5m, max. 108.6m, s.d.: 18.88m) (p-value < 0.0009) and Q3 points (mean:26.6m, min. 4.3m, max: 155.3m, s.d.: 31.30m) (p-value < 0.0001). No significant difference was detected between Q2 and Q3 points (p-value: 0.087) (Fig. 7).

## Discussion

Using harbour porpoise as an example, this study demonstrates that the strengths and weaknesses of theodolites and drones complement each other in positive ways. Drones allow for a detailed assessment of behaviour and life history and collect more precise positional data, but theodolites are faster and cover a larger number of sightings while giving a more complete cover of a full sighting from start to end. The simultaneous use of both methods is recommended to improve the observation studies of harbour porpoises and other coastal marine mammals. Our results can be generalized and put into perspective for smaller cetaceans using a SWOT analysis (Table 4.).

**Table 4:** SWOT (Strengths, Weaknesses, Opportunities and Threats) analysis of theodolite and drones as land-based observation methods for small odontocetes.

	Theodolite	Drone
Strengths	Effective monitoring tools for large marine fauna^3,24,36^ and smaller marine fauna^24^ (present study) Non-invasive, non-disturbing methods^3,24,37^ to estimate precise geographic positions^37^ and movement parameters calculable (speed, heading, bearing change, surfacing intervals) in a comparably large area^3^ Easily transported and flexible data acquisition timing, low cost associated with data collection compared to established methods^24,38^, requires little training	
	Allows to describe coarse behaviours, collect distribution and relative abundance data^14^ Inexpensive for long term use^4^ Weather resistant and operable in windy and rainy conditions with reduced accuracy^39^ Easy to use, low battery consumption and high data storage capacity, easy download and processing of data	Highly accurate location data can be extracted using CetTrack, Pix4D, Agisoft Metashape^21^ Underwater behaviours can be observed^24,37,40^ Enables access to sites considered inaccessible such as Marine Protected Areas (MPAs) or remote islands^37,40^ when permitted Can be operated safely from small research vessels (< 6m)^40^ Multiple observers can review drone footage allowing for accurate, continuous sampling^37^
Weaknesses	Both methods are weather restricted. Theodolites by the visibility of porpoises and drones by strong winds and rain	
	Limited coverage to animals passing close to shore^39^ Requires an elevated geographically vantage point with unobstructed view of the whole research area^38^. Higher observation points lead to more precise positioning^4^ Increasing error in geographic positions with increasing distance^39^, accuracy is much lower than anticipated (present study) due to the need to operate fast Only surface behaviours can be observed^3,26^ Challenging when studying elusive animals that surface for only seconds at a time, move quickly, range large distances and have an unpredictable moving pattern^3^ Limited when identifying individuals, sex, age, reproductive status or body conditions^3^ Not appropriate to study group behaviours and spatial structures as it allows observing only one animal at a time, and it is hard to continuously track the same animals once it submerged under the water^41^ High initial financial investment^4^	Only non-invasive when used properly^24,42^ Battery technology and associated limited flight times make it currently difficult to cover large areas^38^, making long-term studies difficult^37^ Lithium-polymer batteries can pose a safety risk when they spontaneously overheat while over- or undercharged^21^ Visibility below water is affected by waves, reflections, water turbidity, and objects^36^ Requires large data storage: at 4K resolution: ~ 32 GB per hour^21^ Post video processing requires high investment of time, skills, computer programmes and staff^38^ to derive accurate and meaningful information^43^ Requires training, certificate and insurance to be used Unpredictable surfacing patterns and behaviour render odontocetes a difficult target to follow, particularly in deep or turbid water even for experienced pilots^21^ Biased group size for many animals, limited recording large groups, e.g. common dolphin pods exceeding 100 individuals^25^
Opportunities	Can be complemented with other methods^37^ Potential to replace more invasive monitoring techniques^37^	
	They serve different purposes, and are thus often available for rent or to lend at some academic departments^4^ A large amount of studies have been carried out using a theodolites to observe marine mammals^3^, there are established workflows on how to use it and recommendations and considerations are available on how to increase the accuracy of data collection	Artificial Intelligence (AI) can help to process data^36,38^ Special permits can be requested to fly outside established limits^21^ Drones can be used for a variety of research questions: collect behavioural data, study body conditions, photo-ID, collect samples (breath collection, fecal samples)^23,26,44^ Allows eliminating the research boat bias making it a good tool for shore-based observers^45^ Drone images can be used for identifying the number of neonates, calves and juveniles, identifying sexual maturity, mother/calf pairs, body shape and nutritional conditions^40^
Threats	The accuracy of the geographical position can be influenced by the experience of the observer, the size of the observed species, imprecision in measuring of the elevation of the instrument above the sea level, poor calibration and refraction^39^ sea state, swell and waves^4^ Challenging when working with small inconspicuous cetaceans such as harbour porpoises where surfacing’s are short and can be missed if the observer is looking somewhere else (present study) Collecting drone footage is not always easy (hard to locate and follow the animals)	Common drone airspace rules^38^ and restrictions on where and when they can be flown^36^ can limit drone application Large drones can cause life-changing or lethal injuries when they interact with people^43^, smaller drones can produce cuts and bruises Drones flown continuously during the present study regularly showed misfunction, missions had to be aborted, drones flown back to base due to software or mission failure or batteries drained or malfunctioning. This results in lost research opportunities (present study) Pilot fatigue^21^

### Method comparison

We observed that none of the methods allowed to fully record all sightings observed by plain eye and thus only a part of the information available could be captured. Theodolites performed better in collecting at least some positional data of many sightings, similarly to previous findings^46^, while drones captured more detailed data of fewer sightings. Main reasons for not using drones on every sighting were 1) very short sightings with animals appearing and disappearing quickly, also affecting the success of the theodolite; 2) distant sightings were considered difficult to locate and therfore not attempted to be captured with the drone to maintain deployability for better chances; 3) difficult weather conditions such as strong winds or rain. Over half of the videos were classified as D3, showing that it is difficult to find and film small porpoises in the murky water and low-contrast conditions of the Baltic Sea. D1 and D2 footage further showed a rather small appearance rate of porpoises with only 22.4% porpoise presence in the screened time. Even when porpoises were in the videos, they were not continuously seen in most cases, even in shallow waters up to 10 m. Drones nevertheless allowed recording more than one porpoise at the same time if they are in the same frame while collecting more information than the theodolite, which is restricted to taking positional data of one individual at a time.

It has to be considered that collecting drone footage is not always easy (hard to locate and follow the animals, pilot fatigue), requires a lot of storage capacity to film and store high resolution video footage, that the processing of drone footage includes several steps (storing footage correctly and backing it up, screening of the footage, and post-hoc processing if required such as cutting videos, extracting coordinates etc.) and can thus be very time-consuming, making it an expensive method in terms of labor time. Furthermore, detecting small cetaceans in footage recorded at 100m above the launch site is challenging as the porpoises appear small and the pixel size limits the image resolution^22^. Altitude is thus a trade-off, as lower altitudes reduces the area covered but increase image resolution, improving detection and identification^24,47^. However, in another study where drones were flown at 120m altitude, the detection reliability for harbor porpoises was not significantly affected by altitude^22^. On the other hand, the theodolite has a long-lasting battery and a large storage capacity for its data. Downloading and processing of the data are fast and take little time. Some programs such as Pythagoras^48^ are further available to help collect, manage and analyze data even in real time in the field.

### Group size

Estimating group size in cetaceans is challenging due to fast and unpredictable movement patterns, the time spent underwater, the unknown proportion of a group underwater, etc.^45^. In this study, group size estimates made with the theodolite appeared to be less accurate, especially for large groups with significantly lower group sizes obtained with the theodolite compared to numbers counted in the drone footage. The drone footage is deemed to be more reliable since it shows more animals together and especially allows to detect MCP even when mother and calf swim closely together. A similar result was also observed in dolphin group counts where ship surveys underestimated group sizes compared to aerial photographs^49^ or more recently for boat-based surveys, where counts by plain eye significantly underestimated group sizes of bottlenose dolphins compared to counts conducted on drone footage^40^. The elusive behaviour of porpoises on the surface indicates that the issue should be more pronounced than in *Tursiops*, due to the former’s less active behaviour and smaller body size. The conditions to focal follow animals with the drone are not always convenient, since the water is often murky allowing for only low contrast during cloudy weather or having glare in the image on sunny days. Drone operators thus had to often fly close to the animals (20-30 m) to ensure not losing them. It is possible that at this height not all animals of a group fit in the frame, potentially leading to underestimation in group counts with the drone. Animals were often lost in the video frames, making re-identification challenging, potentially resulting in a low biased group size estimate.

### Time and distance

The theodolite was able to capture the initial part of a sighting while drones took more time to be started and to find the animals, resulting in missed opportunities. Gathering data fast is especially important for investigations targeting the initial moments of sightings or those that have to capture full tracks such as detection function studies^17,50^ or reaction studies to permanent sources of disturbances^51–53^. The theodolite was also more successful in tracking the animal until the end of the sighting while the drone generally lost them earlier. This can also be explained by the fact that theodolites are able to take far out points while drone operators stopped trying to (re-)find animals or launch a new drone when the chances of sighting the porpoises are already low, which is the case when they are further away. The theodolite thus gives a more complete overview of the movement range of the porpoise within the observed area.

### Behaviour

While all cetacean species spend only brief times at the water surface, the harbour porpoise is a particular case due to its elusive and random behaviour^54^ and their small body size. Wild harbour porpoise behaviour thus remains poorly understood^55^. Certain behaviours can be detected by theodolite operators, but are always limited to surfacing events which last only a few seconds making a behaviour categorization particularly challenging. The theodolite operator is mostly not able to assign behaviours to specific individuals or establish relationships within a group. This study has also shown that the theodolite operators sometimes provided unreliable information that was not confirmed with drone footage such as resting behaviour. It was nevertheless useful to give early and late information of a sighting in which a drone was not flown and allowed bridging gaps in which drones had to be flown back and re-started due to their limited battery capacity. Theodolite observers can reveal general patterns of behaviours and have been used in many occasions in the past to study larger, inshore cetaceans such as humpback whales^56^, southern right whales^57^ and gray whales^58^, and to a much lesser degree smaller delphinid species such as bottlenose dolphins^59^. In recent years, technologies that allow recording them under the water surface, have contributed to the description of certain aspects of their behaviour such as mating^60,61^, group hunting^35^ or catching and handling of large fish^55^, to mention a few. This also increases the risk of losing the animals and maintaining the identification of individual animals when a group is being followed. However, when good footage is available, it allows very detailed observations on their behaviour and the advantage of providing footage that can be played back many times and analysed by different observers^37^. Further, drone footage allow measurement of subtle changes in orientation of observed individuals that go unnoticed by theodolite operators^46^. In recent years, this has allowed the description of new behaviours that take place completely underwater. This study for instance detected fish herding carried out by individual harbour porpoises, which has so far only been described as a group hunting strategy^35^. This study confirms that drone surveys are more suitable for behavioural observations of porpoises than theodolites^24,46^.

### Fine scale distribution data

Obtaining precise geographical positional data is essential in target experiments such as estimating the approach proximity of a cetaceans to a net^18,62,63^ or the reaction towards deterrent devices^7,18,19^ and to study the relationship of individuals within a group^41^ amongst others.

Coordinates of porpoise positions can be obtained from both methods, and at close distances they do not differ strongly. While the theodolite only captures surfacings and the path in between can only be interpolated based on two consecutive surfacing points, the drone can sometimes follow the animals underwater. Depending on the interval chosen to extract positional coordinates from the footage, an extremely high spatial resolution of movement data can be achieved. In this study, all sightings occurred within 1.4 km distance to the coast. The D_t-d_ of Q1 points was not affected in this distance range from the instrument, showing that the theodolite can take good position estimations in this distance range. The fact that most points were collected within 300m from the theodolite in this study is likely not due to a limitation of the instrument, but reflects the near shore distribution of the porpoises in this area during the study period.

Altough theodolite points are less precise, they still allow the approximate location of a porpoise to be determined, which is especially interesting for short sightings or distant sightings that are often recorded by drone, for example. Therefore, much of the variation in D_t-d_ is likely attributed to position errors in the theodolite. Those arise due to the fast operation mode necessary especially for close range operations. Pointing, fine adjustment and performing the measurement must be carried out in less than 5 s to allow a general screening of the study area to be carried out again. Anyone who has operated a theodolite knows that this is not always feasible using the ocular. This results in a much larger actual errors in position estimates for theodolites and challenges the accuracy used in some previous studies^17,64^. The error range in this study, when considering the drone-based coordinates as a reference, ranged between 8.3 m (Q1) to 26.6 m (Q3). This accuracy will depend on the calibration of the instrument, accurate stationing, a correct measurement of the theodolite height, the sea level variations in the study area^65^ as well as the experience of an observer, sea state, wind speed and direction, or an error in the positioning of the crosshair on the waterline^15,39^. Calculated theoretical distance error ranges when tracking dolphins from a 56 m high cliff in Portugal, ranged from 0.2 m for points taken up to 100 m from the instrument based on 0.002°gon deviation, and 2.7 m for points taken up to 1 km from the instrument. This could be attributed to the higher vantage point; however, our study shows that also 0.002°gon deviation (the error that the lens is causing only) cannot be realistically achieved in the field. Depending on the distance, a much larger error is always a given. Such error ranges have also been observed when studying the precision of a theodolite compared to an onboard GPS fromresearch vessels in sperm whales and dolphins at distances between 2 and 26 km from the theodolite^39^. Error in measurements of distances between the theodolite and GPS positions ranged up to ~ 6000 m, with error increasing with distance. Factors that influence the precision of the theodolite in the present study include the comparatively low vantage point (20 m) and the fact that tracking porpoises is more challenging due to the smaller size of the animal and less active behaviour on the surface, which makes it difficult to detect and precisely capture with the crosshair.

Operators had the capacity to take theodolite points with highest quality (Q1) on their first days, showing that it requires little training to obtain good results while also showing good judgement in self-assessing the quality, since distance between simultaneously recorded theodolite and drone coordinates (D_t-d_) increased with quality class. The variation in D_t-d_ was not influenced by the difference between sea state 1 and 2 although that assessment may differ for larger sea states and more data. It has to be considered that porpoises are small and elusive, while larger whales are easier to spot and provide cues such as blows which will allow better data collection with both drone and theodolite.

### Considerations and improvements for future studies

This study was carried out in collaboration with dedicated volunteers calling for a thorough check of each protocol after field work by the responsible scientists. Detailed protocols for observation times in a standard format (like calibrated and synchronized time zones and take off height, etc.) can help data processing, since a large amount of the drone footage will not contain relevant information (no sightings). Hence, if theodolite observer and drone pilot communicate well in the field, they can note behaviours that cannot be seen from the coast without the time-consuming screening of drone footage. In the future artificial intelligence could help with these tasks too. In recent years, several studies and reviews have been published on the use of drones to study marine mammals, providing valuable suggestions and protocols which should be considered to help standardize experimental conditions^24,37,46^.

## Conclusion

This study shows that while drones are a new and popular tool, they do not replace theodolites in every respect. When complete track information is required, theodolites are the preferred method. However, our results also show that inferred information, such as surfacing rates and results that rely on highly accurate positional data, such as acoustic detection functions, must be reconsidered using drones. Our results may apply to a all small and inconspicuous odontocetes with a nearshore distribution.

## Electronic supplementary material

Below is the link to the electronic supplementary material.

Supplementary Material 1

## Data Availability

Data supporting this study are available as supporting materials. It will be made available through OpenAgrar but is currently under revision process.
