# Supplementary material for: Performance of theodolites versus drones in land-based studies of marine mammals
Source: Sci Rep. 2025 Jun 25;15:20302. doi: 10.1038/s41598-025-06978-8 (PMC12198396; doi:10.1038/s41598-025-06978-8)
Supplement: Supplementary file 1 — Supplementary Material 1 [file 41598_2025_6978_MOESM1_ESM.docx]

**Supplementary Material**

Group counts of porpoises by both methods (theodolite and drone). The median is the same, but especially when groups are larger the drone counts more animals.


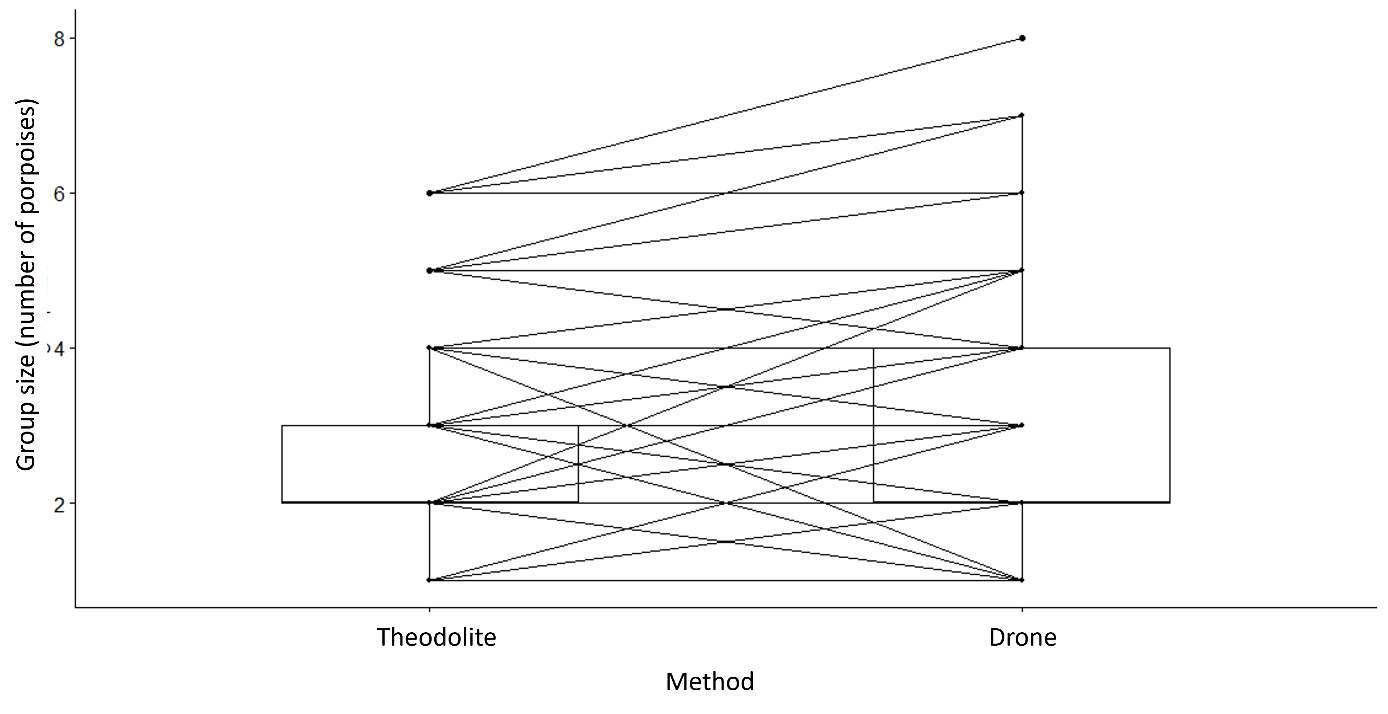


**Time and distance**

Linear regression using a sqrt transformation


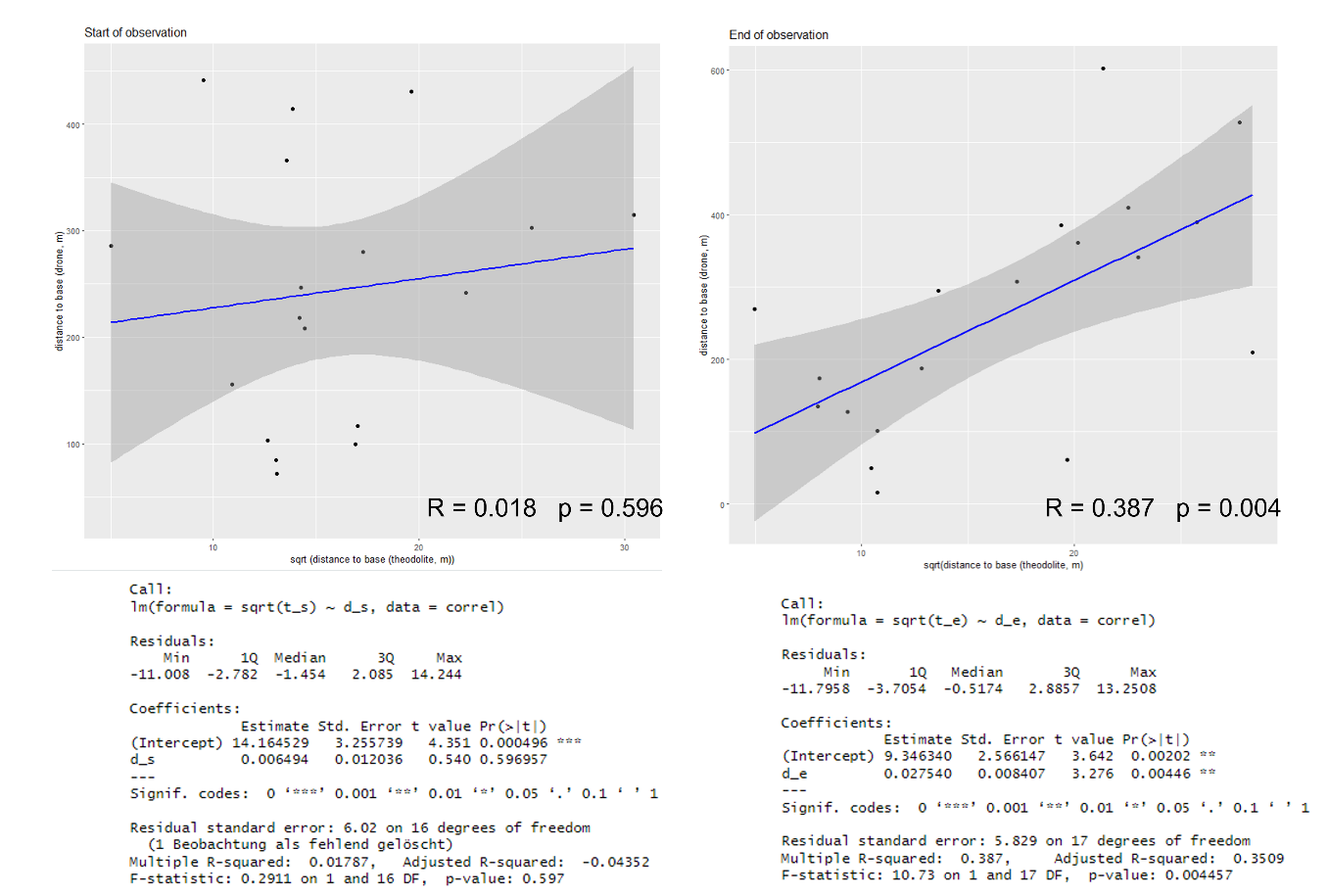


**Field work protocols**

Harbor Porpoise observations (only fill in when there is a sighting)

Date: ___________ Start: ________ UTC End: ________ UTC Net: Yes / No PAL: Yes / No PAL Type: ________

| **ID** | **Time (UTC)** | **Pod size** | **Heading direct.** | **Drome 100m**  **(DMM1 or 2)** | **Drone following (DMM or 2, 3)** | **Quality Drone following (POor/OK/GOod)** | **Theod. Y/N** | **Theod. Start**  **Point** | **Theod. End Point** | **Inside exper. Yes/No** | **PAL Fired?**  **Y / N** | **Time (UTC) PAL** | **Observer** | **Remarks** |
| --- | --- | --- | --- | --- | --- | --- | --- | --- | --- | --- | --- | --- | --- | --- |
|  |  |  |  |  |  |  |  |  |  |  |  |  |  |  |

**Drone quality:** Poor: HP is just for a few seconds on video; OK: HP can be seen on the video with interruptions; Good: HP is on video nearly full time

**Theodolite protocol**

| **Sightings**  **(HP, Net)** | **ID**  **(Nr sighting HP Protocol)** | **Theodolite Point Number** | **Heading direction** | **Start**  **Behaviour** | **Data Quality** | **End Time** | **Observer** |
| --- | --- | --- | --- | --- | --- | --- | --- |
|  |  |  |  |  |  |  |  |
|  |  |  |  |  |  |  |  |

**Sighting**: HP: Harbor porpoise N: Net **Start behaviour**: travelling, feeding, resting, socializing, mating

**Data quality**: 1) The porpoise or its footprint was seen by the observer; high accuracy; 2 = The tracking point is close to the last surfacing; good accuracy; 3) The tracking point is further away of the last surfacing / guessed; medium to low accuracy

**Weather Protocol – Fyns Hoved 2022 (PAL-CE)**

Describe weather conditions at the beginning of each observation and whenever conditions change during an observation. Directions are indicated by radicals in binoculars. Weather has to be given at least ones per hour, please set a timer at the begining as a reminder, please log the Tidal Level at the same time.

| **Date** | **Time (UTC)** | **Sea level** | **Sea state**  **(0 – 4)** | **Wind speed (m/s)** | **Glare direct. (from /to)** | | **% glare cover of study area** | **Cloud cover**  **(1-8)** | **Wind direct.** | **Temp. (°C)** | **Pressure**  **(hPa)** |
| --- | --- | --- | --- | --- | --- | --- | --- | --- | --- | --- | --- |
|  |  |  |  |  |  |  |  |  |  |  |  |

**Drone Protocol**

Describe the aerial footage that was collected during a sighting as well as the mode of operation for the drones used. Fill in details on charging level of the large battery.

| **No.** | **Drone ID** | **Time started (UTC)** | **Time landed**  **(UTC)** | **Battery start** | **Battery end** | **Harbour Porpoise ID (s)** | **Follow** | | **100 m** | | **Operator** | **Is-sues?**  **y/n** | **Remarks (e.g. mother calf pair, PAL remote, issues encountered, solutions)** |
| --- | --- | --- | --- | --- | --- | --- | --- | --- | --- | --- | --- | --- | --- |
|  |  |  |  |  |  |  | **Y** | **N** | **Y** | **N** |  |  |  |
|  |  |  |  |  |  |  |  | |  | |  |  |  |
